# Pregnancy and dialysis

**DOI:** 10.1590/2175-8239-JBN-2020-0028

**Published:** 2020-08-07

**Authors:** Catarina Isabel Ribeiro, Natália Silva

**Affiliations:** 1 Centro Hospitalar de Vila Nova de Gaia/Espinho, Serviço de Nefrologia, Porto, Portugal.; 2 Centro Hospitalar de Trás-os-Montes e Alto Douro, Serviço de Nefrologia, Vila Real, Portugal.

**Keywords:** Pregnancy, Renal Insufficiency, Chronic, Dialysis, Gravidez, Insuficiência Renal Crônica, Diálise

## Abstract

The pregnancy rate of women on dialysis is still very low when compared to that of the remaining population. However, recent years have seen an increase in the success rates of these pregnancies. Among the main precautions that must be taken with pregnant women on dialysis are the maintenance of low levels of pre-dialysis urea, the adequacy of the tension profile, the control of anemia and care to avoid infections, nutritional deficits, changes in phosphorus-calcium metabolism and electrolytic fluctuations. It is also necessary to strictly monitor fetal growth and development. Pregnant women on dialysis have a higher probability of maternal and fetal complications; thus the importance of a multidisciplinary approach among nephrologists, obstetricians and pediatricians. The main objective of this study was to review the literature evidence available on pregnancy on dialysis, on the basic principles of the pathophysiology of pregnant women and their particularities in kidney disease. We will address available treatment options, benefits and risks, anticipating possible future challenges. At the end, we will present a clinical case to illustrate the topic.

## RESUMO

O índice de gravidez de mulheres em diálise permanece ainda muito baixo quando comparado ao da população restante. Contudo, nos últimos anos, o sucesso dessas gestações tem aumentado. Entre os principais cuidados que se deve ter com as grávidas em diálise estão a manutenção de níveis baixos de ureia pré-diálise, a adequação do perfil tensional, o controle da anemia e os cuidados para evitar infecções, *deficit* nutricionais, alterações do metabolismo fosfo-cálcio e flutuações eletrolíticas. O monitoramento rigoroso do crescimento e desenvolvimento fetal é também necessário. Gravidezes em diálise estão associadas à maior probabilidade de complicações maternas e fetais, por isso é de extrema importância uma abordagem multidisciplinar entre nefrologistas, obstetras e pediatras. O objetivo principal do presente trabalho consiste na revisão da evidência literária disponível sobre a gravidez em diálise, sobre os princípios básicos da fisiopatologia da grávida e de suas particularidades na doença renal. Serão abordadas as opções de tratamento disponíveis, as vantagens e os riscos, antevendo possíveis desafios futuros. No final, será apresentado um caso clínico ilustrativo da temática.

## Introduction

Despite the small number of pregnant patients on dialysis, despite diagnostic and treatment advances, this has been a growing reality in recent years. According to the literature, there has been an increase in the success rate of these pregnancies, from 25% in the 1980s to more than 90% today.^1^^,^ 2 ^,^ 3 In the Australian and New Zealand Dialysis and Transplantation Register (ANZDATA), the pregnancy rate was 3.3 per 1,000 patients per year between 1996 and 2008, compared with 0.54 and 0.67 from 1976 to 1985 and from 1986 to 1995, respectively.^4^

Most patients on dialysis have sexual dysfunction, not only in the context of organic disease, but also mentally. Currently, for glomerular filtration rates below 5.0 mL/min/1.73 m^2^, amenorrhea and anovulatory cycles are practically a constant. In addition, decreased libido, polypharmacy and depression are equally prevalent in advanced chronic kidney disease.^3^^,^ 5

However, with technological advances and the experience acquired over the last decades, there has been an improvement in the adequacy of dialysis techniques and pharmacological and nutritional approaches for pregnant women on dialysis, providing a significant increase in quality of life and an increase in pregnancy success rates.^5^

Pregnancies that begin before the dialysis period or in the first year of it have more chances of reaching a favorable term, when compared to those from women who become pregnant after a considerable period on dialysis.^1^^,^ 3 Some authors suggest that this fact can be explained by the greater fertility in women with previous residual renal function.^7^

The diagnosis of pregnancy remains a challenge, mainly because the serum chorionic gonadotropin may be elevated even in the absence of pregnancy. In addition, ultrasound is essential for diagnostic confirmation in these patients.^5^^,^ 6

Both hemodialysis and peritoneal dialysis can be substitute therapy options in pregnancy. There are studies with similar outcomes with both techniques.^4^^,^ 8 Despite all the progress made in this field, most newborns are premature, and maternal-fetal complications, such as gestational hypertension, anemia, infections, polyhydramnios and fetal growth restrictions are frequent.^8^

This is controversial, with no consensus, which is the target of joint meetings of nephrologists, obstetricians and pediatricians. This study aims to make a theoretical approach to this topic, presenting the case of a pregnant woman on dialysis. To start, we will address the pathophysiology and complications of pregnant women without kidney disease, with subsequent approach of renal function replacement techniques during pregnancy.

## Materials and Methods

We searched for clinical guidance standards, review articles, systematic reviews, meta-analyzes and randomized controlled clinical trials on Medline, evidence-based medicine sites, index of Portuguese medical journals and bibliographic references of the selected articles published between 1990 and 2018, in Portuguese, English and Spanish, using the keywords (MeSH terms) “chronic kidney disease”, “dialysis” and “pregnancy”. We excluded those articles that did not address the topic of dialysis in pregnancy/ those written in a language other than those mentioned; and clinical cases without review of the topic. We included 21 papers with the aforementioned criteria.

At the end, we present a real clinical case illustrating this topic.

## Theoretical Basis

### Kidney Physiology in Pregnancy

Pregnancy is one of the most important periods of a woman’s life, and it causes several changes to the body, so that the fetus develops in a healthy way, and delivery takes place under ideal conditions. There are numerous anatomical and functional changes, and the hormonal state has a huge impact on the various organs and systems, the kidney is no exception.^1^^,^ 5

During the gestational period, the kidneys undergo changes: there is a 30% increase in renal volume; from 1.0 to 1.5 cm enlargement in renal bipolar diameter; and 50-80% increase in renal blood flow, especially in the first trimester. Proteinuria is normal, due to the increased glomerular filtration rate and the increased permeability of the glomerular basement membrane. In pregnant women, because of this physiological excess of protein excretion, the reference value is different from that of the remaining population; and proteinuria is considered normal up to 300 mg/24 hours.^3^^,^ 7 ^,^ 9 In the beginning of pregnancy, women experience an increase in urination frequency, mainly due to hormonal factors, and at the end of pregnancy this frequency also increases, but for mechanical reasons. A normal pregnancy generates an average weight increase of 10 to 14 kg, corresponding to about 9 liters of water in the body. This increase in circulating volume leads to a state of hemodilution, with a physiological increase in cardiac output.^9^

### Hemodialysis *Versus* Peritoneal Dialysis

For pregnant women with chronic kidney disease who need dialysis, two modalities can be offered: hemodialysis and peritoneal dialysis.^9^^,^ 10

In the current scenario, both therapies have advantages and disadvantages (Table 1). The treatment modality should not be changed with the onset of pregnancy; however, if urgent start is necessary, it may be easier to start with hemodialysis, since the insertion of a Tenckhoff peritoneal dialysis catheter in any period of pregnancy increases the risk of miscarriage.^8^^,^ 9 The pregnancy rate is lower among patients on peritoneal dialysis compared to those on hemodialysis. In an analysis of registry data including 6,230 women aged 14 to 44 years (1,699 on peritoneal dialysis and 4,531 on hemodialysis), 2.4% of patients on hemodialysis became pregnant over a period of four years versus only 1.1% of patients on peritoneal dialysis. We found a similar result from an analysis of ANZDATA data, in which 2.54 pregnancies per 2,000 patients per year occurred among hemodialysis patients versus only 1.06 among peritoneal dialysis patients. We do not know the reasons for the difference in results between the two modalities.^4^^,^ 10

**Table 1:** Hemodialysis and peritoneal dialysis pros and cons during pregnancy

Hemodialysis	
**Pros**	**Cons**
Less dietary restrictions	Worse metabolic control (intermittent dialysis)
Less water restriction	Higher risk of hemodynamic instability
Less overload using the technique	Need for hypocoagulation
	Lower autonomy
**Peritoneal dialysis**	
**Pros**	**Cons**
Better metabolic control (continuous dialysis)	Higher risk of infectious complications ¥
Lower risk of hemodynamic instability	Higher risk of non-infectious complications ¤
Higher degree of autonomy	More difficulty managing volume
No need for anticoagulation	Higher % of intrauterine growth restriction
Preserving residual kidney function	Increase in the frequency of exchanges ŧ

¥ Peritonitis/peritoneal dialysis catheter tunnel infection/outlet orifice infection

¤ Peritoneal dialysis catheter dysfunction caused by obstruction/shifting or associated pain

ŧ Volume increase is not tolerated, especially in the last trimester

The ideal option is not yet clearly defined and may never be. This stresses the relevance of the care team’s clinical experience, and the need for a strictly individualized assessment, with priority for the well-being of the mother and the future baby.

### Pregnancy Timing

#### Before Dialysis

Successful pregnancies are more frequent in women with residual kidney function.^7^ This may be one of the main factors behind the better pregnancy outcomes of women in the pre-dialysis phase or at the beginning of renal function replacement therapy. Borgan et al. described a success rate of around 80% in women who became pregnant before hemodialysis, and 50% in those who were already under treatment.^4^^,^ 11 Other studies also show that the survival of live births is 30% higher when pregnancy occurs in the initial phase of dialysis.^12^^,^ 13 The high rates of abortion before 20 weeks of gestation, in women previously on dialysis, explain this lower rate of overall survival.^7^^,^ 14

In a patient with advanced chronic kidney disease, pregnancy exacerbates some changes, leading to a faster decline in kidney function and causing the need to anticipate the start of renal replacement therapy. The mechanisms that can be associated with this decline are diverse and include poorly controlled arterial hypertension, proteinuria and urinary tract infections.^15^

#### During Dialysis

When compared to hemodialysis, peritoneal dialysis enables a more stable uterine environment, without large fluctuations in blood volume, solutes, electrolytes and blood pressure. It is also associated with higher hemoglobin values, with no need for systemic heparinization.^14^^,^ 16 There is also the possibility of using the peritoneal route for medication administration, such as insulin and magnesium sulfate. Possible complications are catheter obstruction, increased intra-abdominal pressure and infections, such as infection of the outlet orifice, tunnel infection and peritonitis associated with peritoneal dialysis.^4^^,^ 12 ^,^ 15

In turn, hemodialysis can cause severe hemodynamic changes due to the rapid volume depletion, with an important change in blood pressure that increases placental flow reduction. In addition, abrupt reductions in serum urea concentration compromise fetal balance. The weekly time during which pregnant women must undergo hemodialysis can vary, and the medical prescription must take into account the maintenance of stable conditions in terms of blood volume, blood pressure and interdialytic weight gain.^1^^,^ 7 ^,^ 11

### Renal Replacement Therapy Adjustment

To improve the dialysis adequacy and tolerability for pregnant women, several authors have suggested changes to the renal replacement therapy (Table 2).^8^^,^ 17

**Table 2:** Recommendations in dialysis prescription during pregnancy

Dialysis intensification	
**Hemodialysis**	
◦	Increase in dialysis duration to 24 to 36 hours per week ¥ (5 to 7 sessions/week)
◦	Keep Predialytic urea levels < 30-50 mg/dL
◦	Dry weight increase in 300-500 mg weekly in the 2nd and 3rd trimesters
◦	Minimum needed heparinization. Cumarin anticoagulants are contraindicated
◦	Use of biocompatible membranes
◦	Avoid hypocalcemia. Recommended potassium levels in the dialysate between 3.0-3.5 mmol/L. Need for weekly electrolyte monitoring
**Peritoneal dialysis**	
◦	Increase the exchange frequency and reduce the volumes (usually <1.5L)
◦	Adding COPD and APD to optimize the technique
**Blood pressure control**	
◦	Target blood pressure after the dialysis session: 140-120/ 70-90 mmHg
◦	Objective volume monitoring, if possible (bio impedance option)
◦	Avoid hypotension and volume depression episodes
◦	ACEI, ARA II and minoxidil are contraindicated
**Anemia**	
◦	Increase in ESA dose to maintain the hemoglobin levels of 10-11 g/dL and the hematocrit between 30% and 35% (a 50-100% increase is usually necessary)
◦	Iron supplementation to maintain transferrin saturation above 30%. Oral supplementation is usually not enough, and intravenous administration may be necessary
**Phosphorous-calcium metabolism**	
◦	Avoid hypocalcemia and hyperphosphatemia. Weekly monitoring and calcium carbonate 1-2 g/D supplementation are recommended, if indicated
◦	Avoid hypercalcemia after the dialysis. If needed, adjust dialysate calcium (recommended dialysate calcium dosage of 1.50 mmol/L)
◦	1,25-di-hidroxivitamin D (calcitriol) is recommended in cases of primary hyperparathyroidism or vitamin deficiency
Acid-base disorders	
◦	Avoid metabolic acidosis
◦	Lower doses of bicarbonate are recommended in the dialysate (25 mmol/L), because frequent hemodialysis may increase the risk of metabolic alkalosis
**Nutrition**	
◦	Supplementation with folic acid and Complex B vitamins
◦	Increase protein uptake to 1.8 g/kg/D of the pre-pregnancy weight + 10-20g/D
◦	Increase the caloric uptake to 35 Kcal/kg of the weight during pregnancy + 300 Kcal/D

¥ The recommended dialysis time per week varies in the literature, and there was a benefit if higher than 20h and higher than 36h. Less than 20h/week was associated with worse *outcomes*.^3^ COPD – Continuous Outpatient Peritoneal Dialysis APD – Automatic Peritoneal Dialysis ACEI – Angiotensin Converting Enzyme Inhibitors ARA II – Angiotensin II Receptors Antagonists ESA – Erythropoiesis Stimulating Agent

In peritoneal dialysis, one should decrease the volume infusion 1.5 L, with a larger number of exchanges. Some authors also consider an eventual association of outpatient continuous peritoneal dialysis with the automatic one.^3^^,^ 12 ^,^ 15

There are also numerous changes to consider in hemodialysis. Among them, dialysis intensification of for at least 24 hours a week (with six to seven sessions per week), and a dry weight increase of 0.3 to 0.5 kg every 7 to 10 days from the 20th week of gestation. One should also choose low flow and low efficiency hemodialysis; use biocompatible dialyzers (preferably steam sterilized), dialysate with potassium 3.00 mmol/L, calcium 1.50 mmol/L and lower bicarbonate (25 instead of 28-32 mmol/L); and administer the lowest possible dose of unfractionated heparin; although it does not cross the blood-placental barrier.^16^^,^ 17 It is important to regularly review the dry weight, volume status and tension profile, as well as to avoid excessive inter-dialysis weight gains.^3^^,^ 7 ^,^ 15 The main objective is to cause the least possible hemodynamic instability, considering that for every registered hypotension there is a risk of placental hypoperfusion and severe fetal distress. During the gestational period, the women should be moved to in-hospital hemodialysis (and not in the outpatient center), to have better clinical surveillance, as well as better cooperation among medical specialists, if necessary.^8^^,^ 10 ^,^ 16

### Medication and Diet Adjustment

As with other patients using dialysis, controlling anemia, arterial hypertension and nutritional status are of great importance.

#### Anemia

As far as anemia is concerned, the attending physician should administer folic acid twice as often as usual, to minimize fetal changes in the neural tube. Erythropoietin is also recommended, together with intravenous saccharide ferric oxide, and it is pharmacologically safe in pregnancy and breastfeeding.^3^^,^ 5 ^,^ 11 During pregnancy, there may be a relative resistance concerning erythropoietin; thus, one should increase the dose in 50 % to 100%. Hemoglobin values should be kept above 10 g/dL; hematocrit, between 30 and 35%; and transferrin saturation higher than 30%. Whenever possible, one should avoid the appearance of symptoms resulting from anemia and the need for transfusion support.^9^^,^ 12 ^,^ 18

#### Arterial Hypertension

To control arterial hypertension in pregnant women, it is necessary to avoid the use of renin-angiotensin-aldosterone system inhibitors, giving preference to the use of alpha-methyldopa, calcium channel antagonists and beta-blockers. Although controversial, some authors recommend using diuretics with caution, under the not insignificant risk of placental ischemia.^8^^,^ 11 ^,^ 16

#### Nutrition

Avoiding malnutrition is also of great importance, which requires strict nutritional monitoring throughout the gestational period. It is essential to supplement these patients with zinc and water-soluble vitamins to correct deficits, doubling the usual dosage. Protein intake also needs to be increased to 1.8 g/kg, with an additional supplement of 20 grams daily for fetal growth.^13^^,^ 18

Mineral and bone metabolism must be controlled during this period. These women also require greater dietary freedom, to provide for better phosphorus uptake. However, in patients with phosphorus levels above 5.5 mg/dL, and in the absence of hypercalcemia, we recommend supplementation with calcium carbonate in a maximum daily dose of 2 grams; ingestion of the supplement involves weekly monitoring of serum calcium and phosphorus levels. If there is a deficit of 1,25-dihydroxyvitamin D, we recommend the intake of calcitriol, without exceeding the weekly dosage of 1.5 µg. Calcimimetics are not recommended, because the safety of its administration has not yet been proven.^6^^,^ 10 ^,^ 12

### Obstetric Evaluation and Follow-Up

We recommend fetal monitoring with obstetric ultrasound, umbilical artery Doppler flowmetry and cardiotocography. Serum chorionic gonadotropin should not be monitored, despite being part of the tests registered during pregnancy, because of its high probability of presenting false positives in dialysis patients.^4^^,^ 10 ^,^ 19

Ideally, these women require fetal and uterine ultrasound between the eighteenth and the twentieth week; and the attending should monitor for the risk of preeclampsia from 20 weeks onwards.

Some authors suggest the induction of labor from 36-38 weeks, as long as there is fetal lung maturity, given the high risk factors for stillbirth. However, one should only perform a caesarean section according to the formal indications of all pregnant women.^15^^,^ 16

As they present concentrations of urea and creatinine similar to those of their mothers, and given the high risk of complications inherent to neonatal uremia, newborns should be admitted to Neonatal Intensive Care Units.^3^^,^ 8 ^,^ 17

### Main Maternal and Fetal Complications In Dialysis

The maternal complications most frequently identified in pregnant women on dialysis are anemia and high blood pressure. Anemia secondary to systemic hemodynamic status is often of a multifactorial etiology, even when contextualized with chronic kidney disease. In turn, arterial hypertension affects about 80% of patients, with blood pressure values ​​that exceed 140/90 mmHg; of these, about 40% develop severe hypertension, with values higher than 170/110 mmHg, and need three or more drugs for control.^3^^,^ 17 ^,^ 20

Two other conditions, less frequent but not insignificant in severity, stand out: preeclampsia and HELLP syndrome. Preeclampsia corresponds to the appearance of new or pre-existing hypertension with proteinuria higher than 300 mg/dL; in the absence of proteinuria, having renal failure, liver failure, thrombocytopenia, brain or visual changes and pulmonary edema must be considered. The HELLP syndrome integrates the typical hemolytic pattern, with overlapping changes in liver enzymes and thrombocytopenia.^7^^,^ 12 Pregnant women with kidney disease on dialysis are considered at high risk for pre-eclampsia. Low-dose aspirin prophylaxis cause a reduction in preeclampsia in women with moderate to high risk of the disease. However, we need further studies to support its benefit in this population. Maternal mortality is a rare event.^15^^,^ 21

With regards to fetal complications, prematurity is the main one, occurring in about 80% of cases, followed by restriction of intrauterine growth in 36% of cases, fetal distress and polyhydramnios. More than a quarter of newborns are underweight for their gestational age, below the appropriate percentile. Congenital changes are rare, reported in less than 5% of cases.^12^^,^ 18

### Post-Delivery Care

Routine postpartum care for women on dialysis is similar to the treatment used for those with normal kidney function. There are no contraindications for breastfeeding, and it is important to avoid aggressive ultrafiltration, because volume depletion can interfere.^8^^,^ 14 ^,^ 20 ^,^ 21

Many women resume their pre-pregnancy dialysis programs three times a week in the postpartum period, since the recommended numerous sessions during pregnancy are difficult to keep.^7^^,^ 10

### How to Face This Challenge In the Dialysis Center?

A case to illustrate this topic: a 26-year-old woman with chronic kidney disease secondary to class IV lupus nephritis, on a regular hemodialysis program started three months earlier, by arteriovenous fistula, submitted to previous immunosuppression with cyclophosphamide (cumulative dose 6.5 grams ), mycophenolate mofetil and steroids. No other relevant medical history. Previous Gestations: II, Para II, with a first healthy pregnancy and a live birth at term, and a current unplanned pregnancy, diagnosed at 16 weeks with ultrasound confirmation.

Intradialytic therapy was readjusted and intensified, with an increase in the number of dialysis sessions to five weekly 4-hour sessions, without heparinization (using frequent extracorporeal circuit washes), and correcting the dialysate concentrations of potassium and bicarbonate to 3.0 and 20 mmol/L, respectively.

We paid particular attention to the volume status and dry weight adjustments, with regular reviews to avoid excessive ultrafiltration; from the 2nd quarter onwards, there was an increase of 300 mg every week, on average.

In order to maintain hemoglobin levels around 10-11 g/dL, there was a need to increase the dose of recombinant human erythropoietin by 50%. We also reinforced the nutritional supplementation, taking into account the nephrotoxicity and teratogenicity of some drugs that the patient was taking (such as angiotensin-converting enzyme inhibitors and calcimimetics), we also optimized the treatment of arterial hypertension and mineral and bone disease. Although there was an expected reduction in blood pressure values during the 2nd trimester, a posteriori they presented a new increase, which motivated the initiation of antihypertensive therapy with alpha-methyldopa. With suspended calcimimetics, under 1.5µg calcitriol per week and with adequate levels of phosphoremia without using chelators. We also achieved a reasonable control of secondary hyperparathyroidism.

However, the patient did not regularly comply with the recommendations, especially regarding attendance to dialysis, and she progressed with a pre-eclampsia, which led to a cesarean section in the 28th week of gestation. She had a preterm delivery, of low birth weight for the gestational age, without apparently identified gross malformations or deficits; needing to stay in the Neonatal Care Unit for 6 weeks. The baby had a good overall progress under corticotherapy, to accelerate fetal maturation.

The mother had an uneventful postpartum period, resuming the regular hemodialysis program with three 4-hour sessions a week, with strict blood pressure monitoring in the first months of puerperium.

As in the case presented, and despite some non-compliance by the patient, the pregnancy of women with end-stage renal disease may actually be possible. This fact is probably due to the improvement in the quality of dialysis materials and techniques, to the increasing knowledge and pharmacological access and to a greater awareness of the nutritional needs and deficiencies of that period. To obtain a successful outcome, it is paramount to manage the Peripheral Hemodialysis Unit and the State Hospital, with the support and collaboration of specialists from different areas. Particularly in the present case, and given the difficulties presented by the patient to comply with some of the recommendations, the authors recognize the need for adding psychologists to the team to better care for the pregnant woman on dialysis and her immediate family.

## Conclusion

Successful pregnancy is possible in women of childbearing age under replacement therapy for kidney function. In the last decades, the dialysis treatment optimization has helped increase the rate of live births; however, these pregnancies have a higher risk of fetal and maternal complications when compared to pregnancies of women who are not on dialysis.

Today, we know that hemodialysis and peritoneal dialysis can be replacement therapy options to be offered to pregnant women. In the literature, the main measures to be taken include increasing the weekly dialysis time, maintaining low levels of pre-dialysis urea, controlling anemia and preventing infections, tension and electrolytic oscillations.

In order to obtain the best outcomes, the multidisciplinary care provided by nephrologists, obstetricians, pediatricians, nutritionists and psychologists is becoming increasingly important.
